# Applying Computational Fluid Dynamics in the Development of Smart Ripening Rooms for Traditional Cheeses

**DOI:** 10.3390/foods10081716

**Published:** 2021-07-23

**Authors:** Nuno Alvarenga, João Martins, José Caeiro, João Garcia, João Pássaro, Luis Coelho, Maria Teresa Santos, Célia Lampreia, António Martins, João Dias

**Affiliations:** 1 Unidade de Tecnologia e Inovação, Instituto Nacional de Investigação Agrária e Veterinária, Avenida da República, Quinta do Marquês, 2780-157 Oeiras, Portugal; nuno.alvarenga@iniav.pt (N.A.); pedro.louro@iniav.pt (A.M.); 2 Geobiosciences, Geobiotechnologies and Geoengineering (GeoBioTec), Faculdade de Ciências e Tecnologias, Universidade Nova de Lisboa, 2829-516 Caparica, Portugal; 3 Instituto Politécnico de Beja, Rua de Pedro Soares, 7800-295 Beja, Portugal; joao.martins@ipbeja.pt (J.M.); j.caeiro@ipbeja.pt (J.C.); t.santos@ipbeja.pt (M.T.S.); celia.lampreia@ipbeja.pt (C.L.); 4 Instituto Politécnico de Setúbal, Estefanilha, 2760-761 Setúbal, Portugal; joao.garcia@estsetubal.ips.pt (J.G.); joao.passaro@estsetubal.ips.pt (J.P.); luis.coelho@estsetubal.ips.pt (L.C.); 5 CINEA-IPS, Energy and Environment Research Centre, IPS Campus, Estefanilha, 2760-761 Setúbal, Portugal

**Keywords:** cheese ripening, Internet of Things, Industry 4.0, computational fluid dynamics

## Abstract

Traditional ewe’s cheese producers face certain challenges caused by fluctuating environmental parameters inside the ripening room, which lead to lack of homogeneity in the final product. The present research discusses the application of computer fluid dynamics for simulating the distribution of environmental parameters, predicting the airflow pattern, and identifying critical areas where such parameters could cause reduced cheese quality. A new monitoring system was developed including presence sensors, temperature and humidity dataloggers, pneumatic actuators, microcontrollers, and microcomputers connected remotely for control, data visualization, and processing. The validation of the computer simulation and monitoring system was made with a batch of 40 ewe’s cheeses distributed in three different zones inside a prototype ripening room and ripened for 35 days. At 35 days, a physical, chemical, and microbiological characterization of cheeses was made for evaluation of the influence of environmental conditions on cheese quality. The comparison between simulated and local measurements showed close agreement, especially concerning air velocity inside the stacks of cheese. The results of Pearson’s correlation analysis and PCA concluded that temperature affected the appearance of the rind, hardness, number and area occupied by holes. Humidity affected a_w_ and mFeret. Air velocity affected pH and the circularity of gas holes.

## 1. Introduction

Advances in a set of modern technologies and the appearance of new hardware and software platforms, namely sensors and actuators, data communications, low-cost computing platforms, complemented by cloud computing and artificial intelligence open the possibility of a plethora of new applications generally referred to as the “Internet of Things” (IoT), which is also paving the way for a new industrial revolution termed Industry 4.0. One of the main industries that can benefit from these technological advances, essential for human survival and human wellbeing is the food industry [1]. Computational fluid dynamics (CFD) is a powerful tool for simulating physical processes through numerical analysis and data structures with features defined by boundary conditions which would otherwise require experimental setups which, in turn, are expensive to build and operate. With a CFD tool, phenomena such as fluid flow, heat exchange, or gas species processes (among many others) can be studied and modeled separately or together to simulate a variety of complex systems. Such systems could be natural processes or even food altering/conservation processes from the food industry. Although this numerical analysis has existed for some time, only relatively recently has it begun to be applied in the food industry, either to optimize or to study methods that have long been used empirically but are not completely understood, or to study how specific parameters can impact the results. Some examples in the food industry include applications with evaporative cooling and drying that can be applied to several kinds of meat, vegetables, fruits, cheeses, and cured meats [2,3]; chilling conditions for the conservation of food products and air distribution [4,5], drying and heat release during ripening of fruit and vegetal products [6,7], study of the fermentation processes in different food products [8,9], research and development of new metrics for the assessment of efficient food processes [10], characterization and optimization of food processes along with improvements in product homogeneity and energy waste [5,7,9,11], and influence of storage facilities on harvested products [12].

In Portugal, there is a longstanding tradition of using raw sheep milk and vegetable coagulant in cheesemaking. Consequently, Portugal is the European country with the highest number of cheeses using Protected Designation of Origin (PDO) and coagulated with *Cynara* spp. infusion. Inside Alentejo province there are three PDO cheeses using aqueous extract of *Cynara cardunculus* L. flower (Serpa, Évora and Nisa cheeses) and one Protected Geographical Indication (PGI) cheese where the coagulant can be vegetable or animal rennet (Mestiço de Tolosa cheese) [13]. The cheese making process starts with the filtration of whole raw sheep milk heated to 30–31 °C inside steel vats. Following this, salt and *C. cardunculus* aqueous extract is poured in for coagulation. Such a process usually takes around 40 min, after curd is cut and left until conclusion of syneresis, around 15 min. The whey is then drained, and the curd is poured into plastic or metal molds, lined with a cloth, and filled manually. The pressing stage takes around 45 min and is completely manual. After around 8 h, cheeses are demolded and ripened for about 30 days [14] in two different main stages of 15 days each. Therefore, the present study was set up to evaluate the influence of the environmental conditions inside a prototype for a ripening room, based on CFD and remote control, on physical, chemical, and microbiological parameters of sheep’s cheese after ripening time.

## 2. Materials and Methods

### 2.1. Cheesemaking

**Figure 1:**
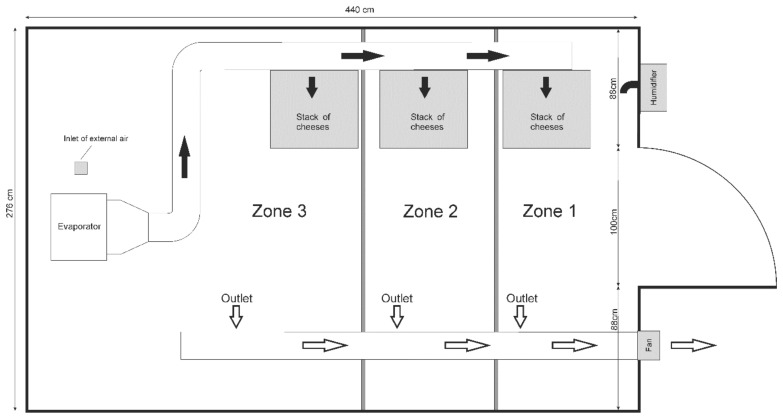
Top view of prototype ripening room.

**Figure 2:**
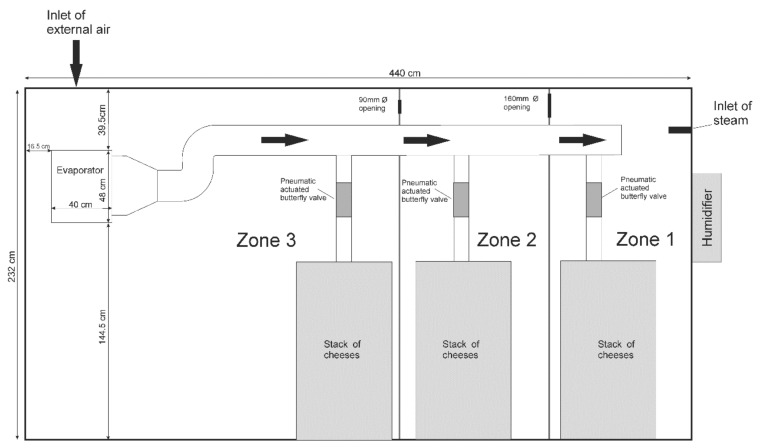
Side view of prototype ripening room.

A batch of 40 semi-hard cheeses was produced using 70 L of raw sheep milk, coagulated with aqueous extract of *Cynara cardunculus* L. flower (0.25 g/L of milk), at 30 °C for 60 min. Then, curd was cut into pieces around 25 mm, whey was separated from the curd, and curd was collected and pressed inside plastic molds. Each cheese presented 86 mm diameter, 53 mm height and around 209 g weight. Cheeses were divided in three batches of 10 units, placed in metal racks in three different zones (Zone 1, Zone 2 and Zone 3) and ripened for 35 days. The dimensions of the prototype ripening room were 440 cm length, 276 cm width, and 232 cm height (Figure 1 and Figure 2).

The refrigeration system included an evaporator installed at the back wall of the prototype (Figure 2), closer to the compressor located externally and using refrigerant R-404 A. An inox funnel was placed at the outlet of the evaporator to conduct the air into a 200 mm Ø PVC duct for distribution to Zones 1–3 through a set of 10 mm Ø circular openings in a 100 mm Ø PVC duct behind the stack of cheeses (Figure 2). Pneumatically actuated butterfly valves (1.1 bar) control the passage of air into the different zones, based on the information provided by a matrix 4 × 4 of presence sensors. The suction of air from each zone was made by a 150 mm Ø (Q = 200 m^3^/h) fan placed externally in a 200 mm Ø PVC duct placed along the ceiling, with three rectangular openings with 700 × 150 mm size each (Figure 1).

### 2.2. Instrumentation of the Ripening Chamber

The data acquisition and control system of the ripening room adopted an IoT architecture whereby a system with sensors, actuators, low-power microcontrollers and microcomputers was connected to the Internet for control and data visualization and processing. The parameters considered for monitoring of cheese ripening included temperature and relative humidity (sensor DHT22/AM2302), air velocity (F660, DegreeC), and presence sensor of cheeses inside Zones 1–3. The monitoring and control system includes the monitoring module and the control module. The monitoring module collected data related to the physical-chemical parameters, while the control module was responsible for the proper adjustments of temperature, humidity and air extraction by activating the compressor of the refrigeration system, humidifier and fan in the outlet duct. The monitoring module is based on a WiPy computational platform (WiPy 3.0-PyCom, 2020), a platform built with the ESP32 microcontroller (Xtensa LX6 microprocessor from Tensilica with a 32-bit CPU architecture). The input and output ports of this microcontroller are particularly suitable for connection to communication systems and data collection. In this case, it features a Wi-Fi communication module for wireless transmission of data collected from the sensors, placed inside the cheese ripening chamber, to the aggregator module outside the room. The individual acquisition modules are associated with static local Internet addresses, assigned by the aggregator module allowing remote access for monitoring and update of the data acquisition software. This computational platform supports the development of software with the MicroPython interpreter version of the Python programming language (“MicroPython-Python for microcontrollers”, 2020), providing several libraries for access to the various hardware subsystems of the WiPy platform.

The aggregator module is built with a Raspberry Pi 3B+ single board computer platform, receiving the information from the data acquisition modules, and storing such data locally. It sends the data through the communications subsystem, via an Internet connection to a remote server. The MQTT Internet protocol was used to transmit data to a remote server (RabbitMQ) (“Messaging that just works-RabbitMQ”, 2020), running on an Arch Linux operating system (“Arch Linux”, 2020). The data is collected using a Node-RED low-code visual programming platform for treatment, visualization and storage in a database (“Node-RED”, 2020). An SSH (Secure Shell) connection in reverse mode made remote access to the aggregator modules possible and, thus, communication with the local data acquisition modules. SSH connections were encrypted and the Internet addresses of each of these local modules were assigned through a DHCP server running in the aggregator module.

### 2.3. CFD Modelling

**Figure 3:**
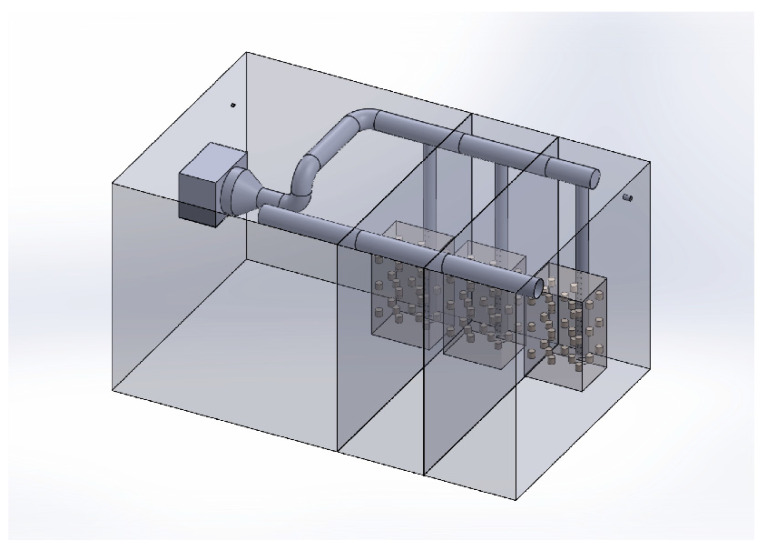
3D model of ripening room.

The geometry of the ripening room was meshed into a hybrid mesh (i.e., including hexahedral and tetrahedral elements) with Ansys Meshing containing a total of 7,500,000 elements and 3,200,000 nodes. A hexahedral mesh was considered for the volume inside the stacks, while a tetrahedral mesh was considered for the remaining volume of the ripening room (Figure 3).

For the solution model, airflow was considered steady, incompressible, and turbulent. The simulation used a k–ε model, as it is a multipurpose, typical model successfully employed in many similar cases, without added computational cost. The first-order upwind discretization scheme was employed because no false diffusion was expected in this model, allowing for a reasonable convergence time. The domain inside the stacks of cheese was considered as a porous medium, coupled with the Ergun equation, according to [15]. Each inlet velocity and temperature (Figure 1) was measured instrumentally with a portable datalogger (model Ami 300 Multifunction, Kimo Instruments, Montpon, France). The inlet air direction was considered normal to the boundary surface. The turbulent intensity for all inlets was 5% and turbulence ratio 10, as per default. The boundary conditions for the room walls were set to adiabatic. The simulated generation of heat and humidity inside the domain of stacks was addressed using a dedicated domain with estimated porosity (56.4%) with values for its inertial and viscous resistance estimated based on the porosity. This domain was employed with two source terms to generate humidity (3 kg/m^3^·s) and heat (10 W/m^3^) from cheeses, based on available literature [16]. The numerical simulations were performed using the software Fluent v17.1 (Ansys, Canonsburg, PA, USA) and for hardware a Xeon E3-1241 3.50 GHz processor, 16 GB DDR3 RAM memory, and GPU NVIDIA Quadro K2200 4 GB graphics card. The convergence residue for simulations was 1 × 10^−3^ concerning continuity, turbulent kinetic energy (k), energy dissipation (ε) and velocity of the x, y, and z vector components, the energy residue value for convergence was 1 × 10^−6^. Computation time was approx. 150 h.

### 2.4. Physical and Chemical Analysis

The water activity values (a_w_) were measured, at 20 ± 1 °C, with a Rotronic Hygropalm HP23-A water activity meter (Bassersdorf, Switzerland). The moisture measurement was performed according to [17], pH was evaluated with a penetration electrode at 20 ± 1 °C (model 691, Metrohm, Herisau, Switzerland). Small amplitude oscillatory measurements (SAOS) were performed, at 20 ± 1 °C, with a controlled shear-strain rheometer (Kinexus lab+, Malvern Instruments Ltd, Malvern, UK) equipped with a 20 mm Ø serrated parallel plate geometry and gap distance of 1 mm, according to [18]. First, linear viscoelastic region (LVR) was evaluated by performing a strain sweep (0.01%–100%) at a steady frequency of 1 Hz. Then, the dynamic frequency sweep was conducted by applying a steady strain of 0.01%, within the LVR, from 0.01 Hz to 100 Hz. Storage (G′) and viscous (G″) moduli at 1 Hz were measured (in kPa). Texture analysis was performed with the texture analyzer TA.XT Plus100 (Stable Micro Systems, Godalming, UK), according to [19]. A 20 mm Ø aluminum probe was used, at a one cycle penetration depth of 20 mm and speed 1 mm/s where hardness (in N) and adhesiveness (in -N.s) were measured. The computer vision analysis of digital images was made according to [20], using software ImageJ 1.52 d (National Institutes of Health, Bethesda, MD, USA), where the following parameters were obtained: number of holes, perimeter, Feret diameter (the maximum distance between any two points along the selection boundary), minimum Feret (the minimum distance between any two points along the selection boundary), circularity, and luminescence (Y). The estimation of the area occupied by gas holes (in %) was performed using the area of gas holes (in pixels) and the total area of cheese sample (in pixels) in the longitudinal cut of sample cheeses. Circularity was estimated using Equation (1):

(1) $$Circularity=4\pi \left(\frac{Area}{Perimeter^{2}}\right)$$

Luminescence (Y) was estimated from the RGB channels according to Equation (2) [21]:

Y = 0.299 R + 0.587 G + 0.114 B(2)

a_w_, pH and moisture were repeated three times per sample. Rheological and image analyses were repeated five times per sample.

### 2.5. Microbiological Analysis

Fractions (10 g) of each cheese under analysis were aseptically homogenized for 120 s, with 90 mL of sodium citrate (GPR) sterile solution (2% *w/v*), in a blender (Seward Stomacher Model 400 Lab Blender, London, UK). The initial suspensions were then decimally diluted with sterile Ringer solution, and duplicate 1 mL or 0.1 mL samples of each appropriate dilution were plated for total or selective colony counting. Media and supplements, unless otherwise indicated, were Biokar Diagnostics (Allone, France). Total aerobic mesophilic bacteria were counted on Plate Count Agar (PCA) incubated at 30 °C for 72 h. Total mesophilic lactic acid bacteria (LAB) on de Man, Rogosa and Sharpe (MRS) agar acidified to pH 5.6 was measured after 72 h at 30 °C. Yeast counts were performed in Rose Bengal Chloramphenicol Agar (RBC), at 25 °C for 72 h. *Enterobacteriaceae* were estimated in Violet Red Bile Glucose Agar (VRBG) after 24 h at 37 °C, where only pink colonies surrounded by a halo of purple precipitate, confirmed as Gram-negative and oxidase-negative, were accounted for. Selective counting of *Streptococcus*, brown, pink, or dark red colonies, was performed in Slanetz and Bartley (SB; Oxoid) agar at 35 °C for 48 h. Transparent and gelatinous colonies, typical of the genus *Leuconostoc*, were estimated on Mayeux, Sandine and Elliker (MSE) agar medium at 21 °C for 72 h. Microbiological analysis was repeated five times per sample.

### 2.6. Statistical Analysis

The average, standard deviation, and 0.95 confidence interval values were determined. Experimental data were subjected to one-way ANOVA (pairwise comparison of means with Scheffé test) in order to compare the average values of different samples. Pearson’s correlation analysis was performed to analyze the correlation between the ripening room environmental conditions (temperature, humidity and air velocity) and physical, chemical and microbiological properties of cheese after 35 days ripening time. The multivariate exploratory techniques, a principal component analysis (PCA), was performed on the results to identify the key parameters describing a global data variability, and to monitor the ripening. Data were analyzed using Statistica 6.0 (StatSoft, Tulsa, OK, USA).

## 3. Results

**Figure 4:**
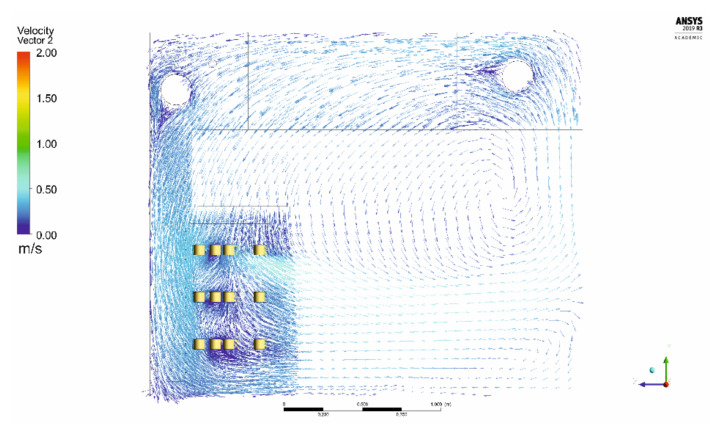
Representation of velocity vector on a vertical cross-section.

**Figure 5:**
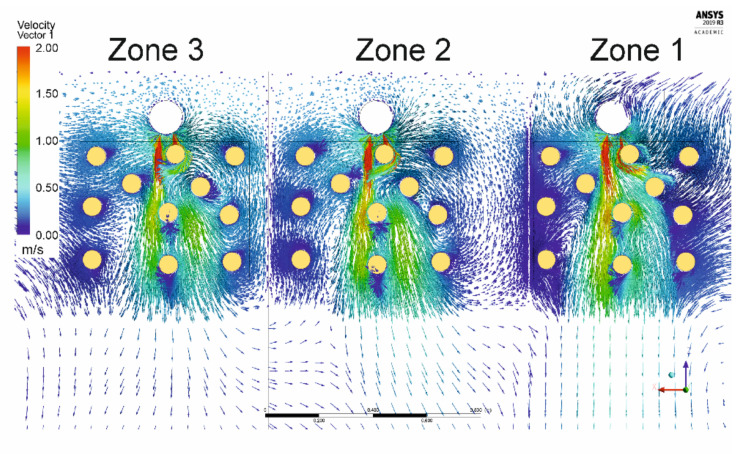
Representation of velocity vector on a horizontal cross-section in Zones 1–3.

The simulation of air velocity vectors, obtained through application of CFD, are represented as colored velocity distribution maps in Figure 4, presenting air velocity pattern in a sample vertical cross-section inside the ripening room, using a denser mesh in critical areas such as inside cheese stacks, close to inlet pipe, or outlet pipe. The simulation predicts a horizontal flow of air, from floor level to 125 cm height, due to the circular openings (left side), moving upwards, due to the proximity of the wall, into the outlet opening in the exhaust duct. From here, the air moves towards the top of cheese stacks and flows downwards, parallel to wall, entering vertically into cheese stacks. Inside stacks, air velocity is affected by the presence of cheese, being reduced to values around 0.25 m/s in the central part and around 0.40 m/s in the side part of the stacks. A stagnation point below the exhaust duct is noticeable, which could cause a higher concentration of water vapor [19] or gases, but as can be observed from Figure 4 and Figure 5, no cheeses were placed in such location.

Figure 5 shows the air velocity pattern through a horizontal plan, at 105 cm height, inside the cheese stacks. It is possible to observe a heterogeneous distribution of air velocity around the cheeses, presenting values around 2 m/s in front of the inlet duct, but below 0.5 m/s in the lateral areas. Noticeably, the prediction of the boundary layer thickness, almost negligible in the frontal area, result of the higher air velocities. On the other hand, cheeses not submitted to such high velocities develop a higher boundary layer, resulting from the surface frictional drag, which affects not only the velocity profile but also the rate of heat flux from and into the cheeses [22].

The average environmental parameters measured inside the ripening room during ripening time are presented in Table 1. The distribution of temperature ranged from 12.3 to 14.6 °C, a consequence of the distance from the evaporator, where lower temperature values were observed in Zone 3, closer to the evaporator (*p* < 0.05). Humidity presented values between 80.8 and 86.8%, higher in Zone 2 (*p* < 0.05). Measured air velocity presented values between 0.14 and 0.20 m/s, with no significant differences between the three zones (*p* > 0.05). The high standard deviation of air velocity was notable, caused by the intermittent operation of the evaporator, which was controlled by the temperature sensor of the ripening room’s compression vapor cycle.

Table 2 presents the average values of physical-chemical parameters at 0 days and after 35 days ripening in Zones 1–3, inside the ripening room. An initial moisture content of 58.9% was observed, decreasing to values between 23.9 and 25.5% after 35 days ripening time, a consequence of the distribution of humidity inside ripening room (Table 1), affecting the desiccation of the cheeses. Previous studies in Évora cheese [19] and Picodon cheese [23] have reported a similar effect.

The observed values on a_w_ were similar to previous study on a similar product [24], where an initial value around 0.95 was observed, decreasing to 0.82–0.84, at 35 days, due to loss of water during ripening time.

The pH presented an average initial value of 6.58, similar to other Portuguese raw milk sheep cheeses [19,25], decreasing to values between 5.30 and 5.44, due to the conversion of lactose into lactic acid by LAB [26,27]. However, no significant differences were observed between zones (*p* > 0.05).

The rheological evaluation included hardness, adhesiveness, G′_1Hz_ and G″_1Hz_ (Table 2). The results on hardness presented values around 5.84 N, at 0 days, increasing to 57.70 N-93.48 N at 35 days (*p* < 0.05), due to desiccation inside the ripening room [26] as observed previously in similar studies [19]. However, other factors may also influence the texture of cheese such as milk composition, moisture, fat content, salt, pH, or extent of proteolysis [26]. However, environmental conditions also present a significant impact [28].

The adhesiveness (-N.s) of cheese presented initial values of 0.96, increasing to 15.89–18.26 after 35 days (Table 2), however no influence was observed due to the location inside the ripening room (*p* > 0.05). The rheological parameters evaluated in the present study presented high standard deviation values (Table 2), also observed in similar works involving cheeses produced with raw milk [26]. Such evidence may result from autochthonous microflora, which will affect proteolysis and lipolysis during the ripening process [29].

Following a similar evolution of hardness, storage modulus G′_1Hz_, presented initial values around 40.1 kPa, at 0 day, increasing to values between 2299.7 and 2823.4 kPa, at 35 days. Viscous modulus G′_1Hz_ presented an initial value around 10.0 kPa, at 0 days, increasing to values between 505.0 and 617.4 kPa, at 35 days. However, both storage and viscous modulus did not show any influence due to the location (*p* > 0.05).

Evaluation of the appearance of the rind of cheeses during ripening was performed through the calculation of luminance (Y_rind_), on a scale from 0 to 255, where 0 represents white and 255 represents black, in a greyscale [21]. The Y_rind_ value at 0 days was 207, similar to previous studies in ewe’s cheese [20] and a consequence of the normal whitish color of fresh sheep’s cheese (Table 2). At the end of ripening, Y_rind_ presented different results according to the location: (i) Zone 1, presented cheeses with the highest Y_rind_, around 223, a significant shift to a brighter appearance during ripening time; (ii) Zone 2, did not present a significant evolution during ripening time, compared with 0 days (*p* > 0.05), and (iii) Zone 3 presented the lowest values of Y_rind_, due to a shift to a yellowish color, as observed previously [19].

Computer imaging was used in the analysis of digital images to evaluate the formation of gas holes during ripening. The percentage of cheese surface corresponding to gas holes presented values at 0 days, around 0.45%, lower than Nisa cheese [20] and a consequence of proper pressurization during the molding stage. After 35 days, all cheese samples increased the area occupied by gas holes to 5.08% at 35 days, where the lowest values were observed in Zone 2 (*p* < 0.05), around 3.08%. The formation of gas holes in such cheese types has been correlated with the presence of specific microflora such as coliforms, yeasts, lactococci [30] or enterobacterias [31], usually part of the autochthonous microflora of ewe’s raw milk [15,32]. The average size per gas hole presented values between 3.28 and 4.43 mm^2^/hole, however with no significant differences during ripening time (*p* > 0.05). Also, circularity presented initial values between 0.32 and 0.42, with no significant differences during ripening time (*p* > 0.05). Also, metric parameters Feret and mFeret did not present significant changes during ripening time or due do the location (*p* > 0.05). These results indicate a consistency of both size and shape during ripening time, where the production of gas during ripening time is limited and insufficient to cause structural changes.

### Microbiological Results

The evolution of the microbiological groups during ripening included total mesophilic bacteria, LAB, *Leuconostoc*, *Streptococus*, *Enterobacteriaceae* and yeasts (Table 2). The total mesophilic bacteria presented 6.17 log, at 0 days (Table 2), increasing to 8.64–8.94 log at 35 days. As in previous studies, LAB presented the highest counts during ripening time, [19,32,33]. At 0 days, LAB presented 4.82 log (Table 3), increasing to values between 8.43 and 8.65 log at 35 days. According to the literature, this groups represents the main microbial group of cheese, forming part of its natural microflora, together with other bacteria, yeasts, and/or molds [34], and is responsible for pH reduction [34,35].

The genera *Leuconostoc* is a heterofermentative group, part of the LAB [27], usually found in Portuguese [32,33] and Spanish [36] cheeses produced with raw milk. The initial count was 2.32 log, increasing to values ranging from 4.25 to 6.82 log at 35 days, slightly lower in Zone 1. The genus *Streptococcus* is also part of the LAB, usually present in “pasta filata” cheeses [37] but also part of the native microflora of Portuguese ewe’s cheeses, such as Évora [32] and Serpa cheese [33]. The initial count was 3.42 log, increasing to values between 6.15 and 6.83 log counts after 35 days. No influence due to the location (*p* > 0.05) was observed.

The *Enterobacteriaceae* is considered an indicator of the hygienic quality [38]. The initial counts were around 4.25 log (Table 2), and no significant changes were observed during ripening time (*p* > 0.05), except for Zone 1, which presented values around 2.39 log after 35 days (*p* < 0.05). However, similar results have been reported in previous studies on raw ewe’s milk cheeses from Spain [31] and Portugal [19,33,39].

The yeasts have been found regularly in traditional Portuguese cheeses produced with raw ewe’s milk [32,40,41]. In the present study, the initial population was 2.46 log units, decreasing during ripening to values between 1.38 and 2.34 log units, less than in similar studies [38,40,41,42]. However, no significant differences were observed during ripening time or due to the location (*p* > 0.05).

The Pearson’s correlation coefficients (r) between environmental conditions and the physical, chemical and microbiological properties of cheese were carried out and the results are presented in Table 4. The results indicate that temperature was the air environmental parameter with the highest impact on the properties, with a significative positive correlation between temperature and color parameters (RGB and Y_rind_) and hardness. Negative correlation between temperature and the number of holes or percentage of holes was observed. Environmental humidity showed a significant positive correlation with a_w_ and mFeret. Air velocity presented a significant positive correlation with pH and a negative significant correlation with circularity.

**Figure 6:**
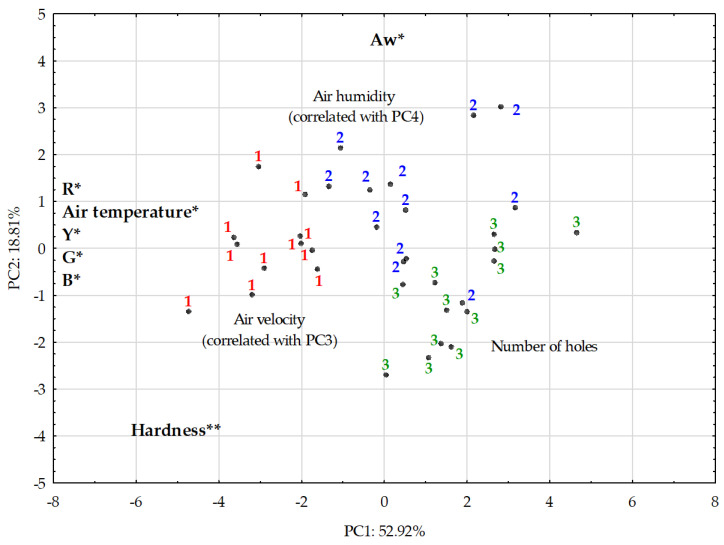
Principal component analysis: PC1 vs. PC2 projection of samples. The most important variables for the definition of the two components are shown on the edge of each axis, indicating the direction in which the value of the parameter increases: * marked values which were considered moderately correlated with the PC; ** marked values which were considered strongly correlated with the PC, following the classification used previously [20]. The plot represents samples ripened in Zones 1–3.

A PCA analysis was carried out on ten parameters (Figure 6), using simultaneous environmental conditions (temperature, humidity, and air velocity) and some cheese characteristics in order to determine the influence of these air conditions in the cheese’s properties (channels RGB, Y_rind_, hardness, a_w_ and number of holes). The similarity map defined by the first two principal components took into account 71.7 % of the total variance (Figure 5). The first component (PC1) by itself condensed 52.9% and the second component (PC2) represented 18.8% of the cumulative variance. The PC1 was explained by RGB parameters, air temperature, and hardness; the PC2 was explained by a_w_ and hardness. The PC1 presented negative correlations with air temperature, image parameters (RGB and Y_rind_), and hardness. The PC2 presented hardness and positive correlations with RGB. The PC2 was negatively correlated to pH and positively correlated to percentage of holes and area per hole.

## 4. Discussion

In this work, the authors presented a new technological approach to the traditional ripening process of ewe’s cheese, including remote monitoring and control, together with optimization of the distribution of environmental conditions using computational fluid dynamics. The need for this new approach results from an evidence in a significant number of traditional cheese factories, namely the effect of heterogenous environmental conditions, caused by different airflow patterns created by the refrigeration system installed in small companies, causing a lack of homogeneity in the final product [43]. As in previous studies [18,26], hardness presented a large capacity for sample discrimination, being the only rheological parameter presenting significant differences between zones inside the ripening room. In fact, cheeses ripened with lower environmental humidity present lower moisture content, harder texture, darker appearance, and larger interior holes [19]. On the other hand, cheeses ripened at higher humidity levels presented a higher moisture content, a softer texture, a lighter appearance, or an excessive presence of molds and yeasts. For example, previous works on Emmenthal cheese ripening concluded that damages to crust formation were caused by differences in the relative humidity of over 10% inside the ripening room [44].

The contribution of computational fluid dynamics is seen as a fundamental tool in modeling and simulating processes for predicting streamlines inside the ripening room and identifying possible stagnation points where humidity may increase above the ideal. To pursue such objective, a ripening room was constructed and divided in three different zones, where a batch of ewe’s cheese was ripened for 35 days. The monitoring system includes cheese presence sensors, temperature and humidity data loggers, pneumatic actuators, microcontrollers, and single board microcomputers connected to the Internet for control, data visualization, and processing. The results of the measured environmental parameters presented a considerable homogeneity of air velocity in the different zones, between 0.14 and 0.20 m/s. The CFD simulation did not present any stagnation point or swirls close to the area occupied by the stacks of cheese, and, additionally, presented an airflow pattern including both horizontal and vertical streamlines inside the volume occupied by cheeses. However, the distribution of the temperature and humidity presented some differences between Zones 1–3, with an impact on cheese ripening. In fact, PCA analysis presented a positive correlation between temperature and RGB color parameters and, consequently, the luminescence of Y rind. Also, a_w_ presented a positive correlation with temperature, however, it may also be affected by a higher environmental humidity. In this analysis it was possible to observe that samples in different regions were separated in the plane. Samples from Zone 1 were subjected to the highest temperature and presented higher color parameter and hardness compared to other samples. On the other hand, PC2 was shown to be more able to separate the samples from Zone 2 and Zone 3. In general, Zone 2 samples presented higher a_w_ values and less hardness than samples from Zone 3. These observations are coherent with previous studies on goat cheese ripening, where the impact of environmental temperature induced a larger effect on proteolysis and lipolysis than humidity [45]. The microbiological analysis presented similar growth to previous works on ewe’s cheese using raw milk [19,33,41], where significant growth of total mesophiles, LAB and yeasts was observed during ripening time. The results presented a similar microbiological profile in the different zones inside the ripening room, leading to the conclusion that microbiota was not affected by the differences in temperature, humidity, and air velocity.

The experimental results in a remote control system demonstrated the possibility of monitoring several environmental parameters in different locations inside the ripening room in real-time. However, the environment inside the ripening room presented somewhat extreme conditions, such as very high humidity levels, which may limit the lifetime of some of the sensors. The implemented system can still be improved with the substitution of some of the sensors by more robust industrial class sensors that are more reliable when operating in such harsh conditions.

## 5. Conclusions

Environmental conditions play an important role during the ripening process of cheese, impacting physical, chemical, and microbiological parameters. The evaluation of cheeses at 0 days was similar to other traditional ewe’s cheese, strongly influenced by the quality of raw milk and the production process. The application of CFD in the prototype ripening room made possible the simulation of airflow through the stacks of cheese, including the formation of a boundary layer near the edges, thus affecting heat and mass transfer. The comparison between simulation and measurements, namely air velocity revealed a considerable agreement in the prediction of distribution inside the prototype, presenting average values inside cheese stacks between 0.14 and 0.20 m/s. Although extreme values such as 2 m/s and 0 m/s could also be observed inside the ripening room. The environmental temperature presented positive correlations with the luminescence of the rind, as stated by Pearson’s correlation and PCA. The temperature also affected hardness, probably due to a higher drying rate. On the other hand, the temperature presented a negative correlation with the number of holes and the area of the surface of cheese occupied by gas holes in the interior. The environmental humidity presented a correlation with a_w_, as expected, due to a lower mass loss during ripening but also with mFeret. Air velocity affected pH and the shape of holes. However, the microflora was not affected either by environmental parameters or the location inside the ripening room. A possible reason may be a short amplitude of values, insufficient to significantly influence the development of microflora during the ripening time. The use of remote monitoring and control of the ripening room allowed a permanent tracking of the environmental conditions in real-time, however, some improvements can be made, namely the selection of more robust sensors better suited for the extreme conditions inside the ripening room, namely the high humidity levels.

## Figures and Tables

**Table 1:** Mean values (±standard deviation) of temperature, humidity, and air velocity under different environmental conditions.

.	Zone 1	Zone 2	Zone 3
Temperature (°C)	14.6 ^a^ (1.4)	13.6 ^b^ (0.4)	12.3 ^c^ (0.7)
Humidity (%)	82.9 ^b^ (3.1)	86.8 ^a^ (4.1)	80.8 ^b^ (2.7)
Air velocity (m/s)	0.14 ^a^ (0.14)	0.20 ^a^ (0.15)	0.20 ^a^ (0.19)

^a,b,c^ Means in the same row marked with different letters are significantly different (*p* < 0.05, *n* = 10, Scheffé test).

**Table 2:** Mean values (±standard deviation) of physical-chemical evaluation of cheeses during ripening under different environmental conditions.

Parameter	0 Days	After 35 Days of Ripening		
		Zone 1	Zone 2	Zone 3
Moisture (%)	58.9 (1.1) ^a^	24.7 (1.4) ^bc^	25.5 (1.3) ^b^	23.9 (1.4) ^c^
a_w_ (-)	0.95 (0.02) ^a^	0.82 (0.01) ^c^	0.84 (0.02) ^b^	0.83 (0.01) ^bc^
pH (-)	6.58 (0.11) ^a^	5.32 (0.15) ^b^	5.30 (0.13) ^b^	5.44 (0.21) ^b^
Hardness (N)	5.84 (1.0) ^c^	93.48 (18.07) ^a^	57.70 (16.41) ^b^	76.82 (21.6) ^ab^
Adhesiveness (-N.s)	0.96 (0.43) ^b^	18.26 (4.51) ^a^	16.68 (3.70) ^a^	15.89 (5.57) ^a^
G′_1Hz_ (kPa)	41.0 (7.3) ^b^	2 693.1 (408.4) ^a^	2 299.7 (530.8) ^a^	2 823.3 (796.0) ^a^
G″_1Hz_ (kPa)	10.0 (1.9) ^b^	569.7 (84.0) ^a^	505.0 (109.0) ^a^	617.4 (151.5) ^a^
Y_rind_ (-)	207 (10) ^b^	223 (3) ^a^	205 (10) ^bc^	196 (6) ^c^
Area of holes (%)	0.45 (0.58) ^b^	3.78 (2.76) ^ab^	3.08 (1.24) ^b^	5.84 (1.90) ^a^
Area (mm^2^/hole)	3.28 (2.72) ^a^	4.33 (1.78) ^a^	3.96 (1.29) ^a^	4.43 (1.27) ^a^
Circularity (-)	0.42 (0.11) ^a^	0.36 (0.06) ^a^	0.32 (0.05) ^a^	0.35 (0.03) ^a^
Feret (mm)	2.84 (1.61) ^a^	3.11 (0.47) ^a^	3.17 (0.53) ^a^	2.82 (0.34) ^a^
mFeret (mm)	1.86 (0.78) ^a^	1.79 (0.27) ^a^	1.86 (0.32) ^a^	1.7 (0.19) ^a^

^a,b,c^ Means in the same row marked with different letters are significantly different (*p* < 0.05, *n* = 10, Scheffé test).

**Table 3:** Mean values (±standard deviation) of microbiological characterization of cheeses during ripening under different environmental conditions.

Group (log_10_ cfu/g)	0 Days	After 35 Days of Ripening		
		Zone 1	Zone 2	Zone 3
Total mesophilic bacteria	6.17 (0.08) ^b^	8.89 (0.45) ^a^	8.64 (0.31) ^a^	8.94 (0.14) ^a^
LAB	4.82 (0.19) ^b^	8.65 (0.36) ^a^	8.43 (0.31) ^a^	8.62 (0.09) ^a^
*Leuconostoc*	2.32 (0.47) ^b^	4.25 (3.21) ^ab^	6.11 (0.16) ^a^	6.82 (0.93) ^a^
*Streptococcus*	3.42 (0.39) ^b^	6.40 (0.72) ^a^	6.15 (0.27) ^a^	6.83 (0.50) ^a^
*Enterobacteriaceae*	4.25 (0.31) ^a^	2.39 (2.15) ^b^	4.26 (0.50) ^a^	4.44 (0.07) ^a^
Yeasts	2.46 (0.51) ^a^	2.34 (1.20) ^a^	1.75 (0.66) ^a^	1.38 (0.09) ^a^

^a,b^ Means in the same row marked with different letters are significantly different (*p* < 0.05, *n* = 5, Scheffé test).

**Table 4:** Pearson’s correlation coefficients (r) between environmental conditions and physical, chemical, and microbiological properties of cheese after 35 days ripening time.

	Temperature	Humidity	Velocity
a_w_	−0.51	0.56 *	−0.48
pH	0.46	0.00	0.79 **
Moisture (%)	−0.15	0.37	−0.45
R_rind_	0.71 **	−0.21	−0.11
G_rind_	0.74 **	−0.21	0.00
B_rind_	0.74 **	−0.15	−0.01
Y_rind_	0.73 **	−0.2	−0.03
Hardness (N)	0.60 *	−0.53	0.54
Adhesiveness (-N.s)	0.03	−0.18	−0.33
G′_1Hz_ (kPa)	0.23	−0.34	0.23
G′_1Hz_ (kPa)	0.01	0.12	0.07
Number of holes	−0.57 *	−0.35	0.10
Percentage of holes (%)	−0.59 *	−0.29	0.02
Area per hole (mm^2^)	−0.21	0.20	−0.18
Perimeter per hole (mm)	−0.07	0.28	0.14
Feret (mm)	0.06	0.47	−0.22
mFeret (mm)	0.09	0.55 *	−0.15
Circularity (-)	−0.14	0.29	−0.62 *
Total mesophiles (log_10_ cfu/g)	0.18	−0.42	−0.19
*Enterobacteriaceae* (log_10_ cfu/g)	0.02	0.24	0.47
LAB (log_10_ cfu/g)	0.14	−0.20	−0.25
*Leuconostoc* (log_10_ cfu/g)	−0.44	0.08	−0.17
*Streptococcus* (log_10_ cfu/g)	−0.17	−0.30	−0.31
Yeasts (log_10_ cfu/g)	0.09	−0.09	−0.31

* marked correlation significant (0.01 < *p* < 0.05); ** marked correlation strongly significant (*p* < 0.01).
